# Surgical Outcomes and Complications of Patients With Acromegaly in Qatar

**DOI:** 10.7759/cureus.92495

**Published:** 2025-09-16

**Authors:** Alaaeldin Ahmed, Tarik A Elhadd, Fatima Al Sada, Zeinab Dabbous, Sirajeddin Belkhair, Ghanem Al Sulaiti, Amr AlhajAli, Ali Msheik, Ali Ayyad

**Affiliations:** 1 Neurosurgery, Hamad Medical Corporation, Doha, QAT; 2 National Diabetes Centre, Hamad Medical Corporation, Doha, QAT; 3 Endocrinology and Diabetes, Hamad Medical Corporation, doha, QAT; 4 Neurosurgery, Weill Cornell Medicine-Qatar, Doha, QAT; 5 Neurosurgery, Hamad General Hospital, Doha, QAT

**Keywords:** acromegaly, densely granulated, hardy classification, knosp, serum igf-1

## Abstract

Background: Acromegaly is a rare endocrine disorder. It results from excess growth hormone (GH) secretion, predominantly due to pituitary adenomas. Endoscopic trans-nasal transsphenoidal (ETT) resection remains the primary treatment modality. Adjunctive medical and radiotherapeutic interventions are required for incomplete remission. This study evaluates the surgical outcomes and complications in patients with acromegaly managed at Hamad General Hospital (HGH), Qatar.

Methods: This retrospective study includes patients diagnosed with acromegaly and managed at HGH between January 2010 and May 2025. Only patients with confirmed acromegaly and available preoperative and postoperative hormonal assessments and imaging were included. Hormonal remission rates, extent of tumor resection, and postoperative complications were the key outcomes.

Results: A total of 45 patients were included; 23 underwent surgery at HGH. The median patient age was 43 years, with a total of 39 males. ETT resection was the sole surgical modality. Postoperatively sustained biochemical remission was observed in most cases at 12-month follow-up with lowered insulin-like growth factor-1 levels (IGF-1). Lower recurrence rates (17.3% vs. 8.88%) and a higher proportion of complete tumor resection were evident in the group of patients operated at HGH. Postoperative pituitary function was preserved in 73.9% of patients with a low incidence of complications such as cerebrospinal fluid (CSF) leaks, diabetes insipidus, and infections. Histopathology revealed a predominance of densely granulated somatotroph adenomas in the HGH group, associated with improved surgical outcomes.

Conclusion: ETT surgery remains an effective primary treatment for acromegaly. Favorable biochemical remission rates and tumor resection outcomes are observed in patients managed at HGH. The predominance of densely granulated somatotroph adenomas in this cohort may have contributed to the observed favorable surgical outcomes. Further large multicenter studies are warranted to validate these conclusions.

## Introduction

Acromegaly is a rare endocrine disorder characterized by excessive secretion of growth hormone (GH), predominantly caused by pituitary adenomas. This pathological overproduction of GH leads to elevated levels of insulin-like growth factor 1 (IGF-1), resulting in progressive somatic disfigurement, organomegaly, and various systemic complications [1-3]. If left untreated, acromegaly is associated with increased morbidity and mortality due to cardiovascular, metabolic, and respiratory complications [4-12]. Early diagnosis and effective management are crucial for improving patient outcomes.

The primary treatment objective in acromegaly is to normalize GH and IGF-1 levels, alleviate symptoms, and prevent long-term complications. Surgery is recommended as the primary treatment of acromegaly [6]. The endonasal transsphenoidal approach surgery remains the cornerstone of treatment for acromegaly [9]. Advances in surgical techniques, including endoscopic approaches, have enhanced tumor visualization and resection rates, improved biochemical remission, and reduced complication rates. Despite these advancements, achieving complete remission can be challenging, particularly in patients with invasive or large macroadenomas. In such cases, adjunctive therapies, including medical management and radiotherapy, are often necessary to control hormone hypersecretion and tumor growth [8].

This study was conducted to evaluate the surgical outcomes and postoperative complications of patients with acromegaly managed at Hamad General Hospital (HGH), a tertiary care center in Qatar, over 15 years (2010-2025). Specifically, the objectives were to: 1) Report biochemical results following endoscopic trans-nasal transsphenoidal surgery, using serum IGF-1 levels at defined postoperative intervals as the principal marker of disease control; 2) Evaluate radiological outcomes, including the extent of tumor resection (gross total vs. subtotal), the definition of residual and complete excision on magnetic resonance imaging (MRI), and the frequency and timing of follow-up imaging to monitor recurrence; 3) Analyze surgical complications such as cerebrospinal fluid (CSF) leaks, new-onset hypopituitarism, diabetes insipidus, meningitis, and vascular or cranial nerve injuries; 4) Characterize the patient population in terms of demographics, comorbidities, and histopathological subtypes (including granulation patterns), and assess their association with surgical outcomes; 5) Provide institutional outcome data from a regional center of excellence, contributing to the global literature on acromegaly surgery and establishing a benchmark for future multicenter comparisons.

## Materials and methods

Study design and methods

This study was designed as a retrospective chart review aiming to describe the clinical characteristics, surgical outcomes, and postoperative complications of patients diagnosed and treated for acromegaly at HGH, a tertiary referral center in Doha, Qatar. The study period spanned 15 years, from January 2010 to May 2025. All patient-related data were extracted from the hospital’s electronic medical records system, operative notes from the Department of Neurosurgery, and longitudinal follow-up documentation maintained by the Endocrinology Department. Data extraction was performed by trained clinical researchers using a standardized data collection form to ensure consistency and reliability.

Inclusion and exclusion criteria

Patients were eligible for inclusion if they had a confirmed diagnosis of acromegaly, established through a combination of clinical presentation (acral enlargement, facial coarsening), biochemical testing (elevated serum IGF-1 levels and inadequate suppression of GH after oral glucose tolerance testing), and radiological evidence of a pituitary adenoma on MRI. Only patients who underwent surgical intervention and had complete preoperative and postoperative hormonal profiles and neuroimaging studies were included. Patients with insufficient medical documentation, absence of follow-up data, or alternative diagnoses were excluded from the analysis.

Outcomes assessed

The primary outcomes of interest included: Hormonal Control: Changes in serum IGF-1 levels preoperatively and at designated postoperative intervals are used as a biochemical marker for disease activity.

Radiological assessment was performed using MRI at baseline (preoperatively), immediately postoperatively, and during scheduled follow-up intervals. Standard pituitary imaging protocols at HGH included T1-weighted sequences (pre- and post-gadolinium contrast), T2-weighted sequences, and dynamic contrast-enhanced studies for evaluation of the sellar and parasellar regions. The extent of resection was defined on postoperative MRI as gross total resection (GTR) when no residual enhancing tumor was visualized, or subtotal resection (STR)when any degree of residual enhancing lesion was present. Radiological recurrence was defined as the appearance of a new enhancing lesion in the sellar or parasellar region after prior documentation of complete resection or as demonstrable interval growth of known residual tumor. Follow-up imaging was typically performed at 3-6 months postoperatively, then annually, or earlier if clinically indicated.

Postoperative Complications: Identification of adverse events following surgical intervention, including CSF leaks, postoperative meningitis, new-onset hypopituitarism requiring hormone replacement, transient or permanent diabetes insipidus, and intraoperative vascular or cranial nerve injuries.

All outcomes were systematically reviewed and recorded, with special attention to temporal patterns of biochemical remission, radiological progression, and delayed complications.

Ethical considerations

This research was approved by the Institutional Medical Research Committee (MRC) of Hamad Medical Corporation (HMC), and the approval number is MRC-01-25-23. Given the retrospective nature of the study and the use of anonymized data retrieved from existing medical records without direct patient contact, the requirement for informed consent was formally waived by the ethics committee in accordance with national and institutional guidelines for secondary use of clinical data.

## Results

Forty-five patients were included, of whom 23 were classed as the HGH group as they underwent their entire treatment at HGH. Twenty-two patients were operated on at different facilities but underwent further treatment, including re-surgery when indicated, at HGH.

**Figure 1:**
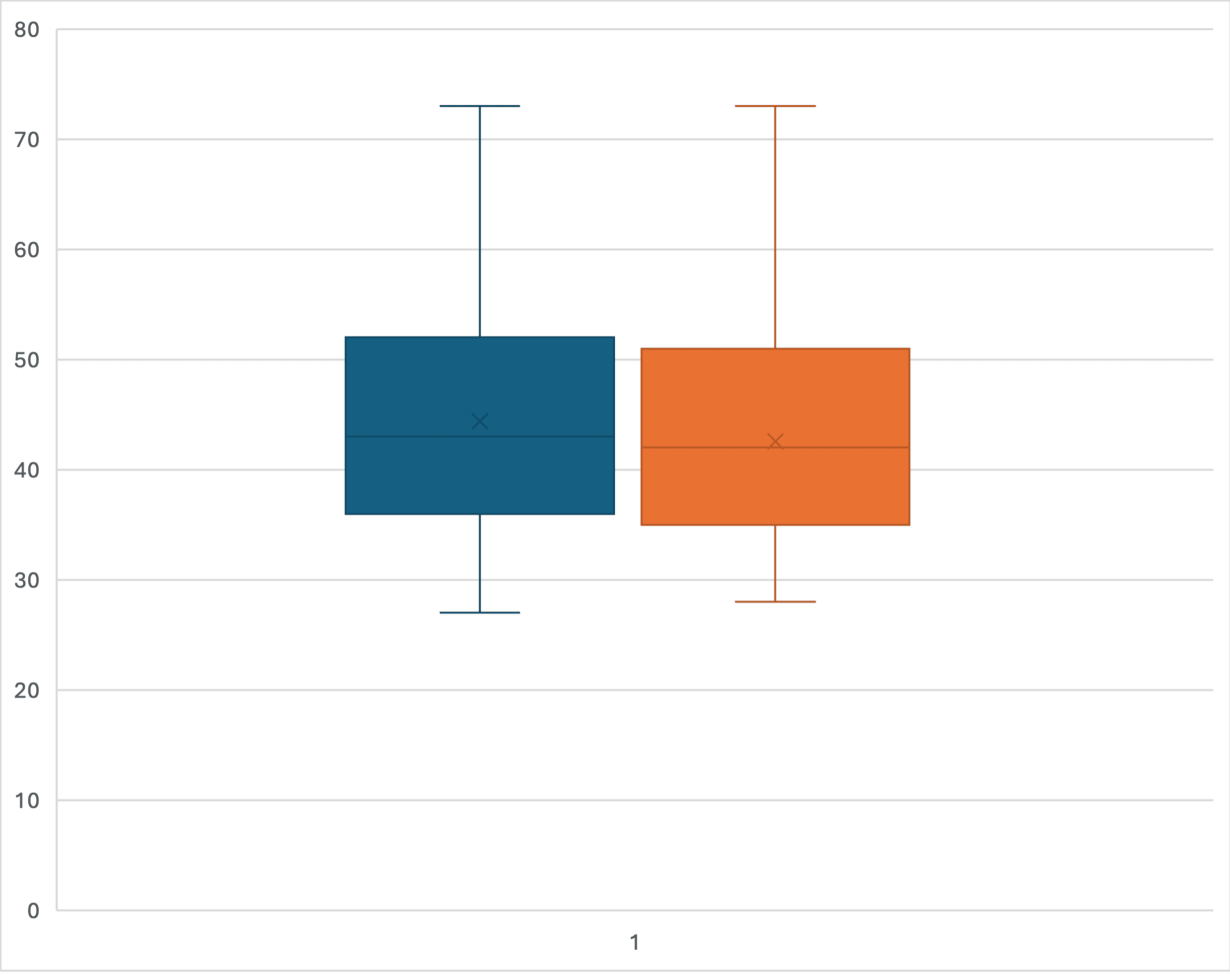
The box plot compares the age distribution of all patients (blue) and the HGH group (orange). HGH: Hamad General Hospital

Both groups have similar median ages (43 years) and interquartile ranges (IQR), spanning from 36 to 52 years in the overall cohort and from 35 to 51 years in the HGH subgroup (Figure 1). The mean age is slightly higher in the overall group (44.38 years) versus the HGH group (42.57 years). The population operated upon outside HGH is marginally older. Hence, the age distributions are largely comparable. 

Males constitute the majority in both categories (39 versus 16 in the overall cohort; 16 versus 7 in the patients in the HGH group). The proportion of the HGH group appears relatively consistent across genders, with approximately 41% (16 out of 39) of all male patients and 44% (7 out of 16) of all female patients undergoing surgery at HGH.

The largest subgroup is South Asian, with 13 total patients (7 in the HGH group). Non-Qatari Arabs are the second largest group with 11 total patients and 8 receiving surgery at HGH. Qataris and Africans each have eight patients overall, with 3 and 2, respectively, receiving surgery at HGH. Smaller groups include Europeans (2 total, 1 operated) and Eastern Asians (3 total, 1 operated).

To report the preoperatively and postoperative findings, proportions (incidence of event/total of cohort) and/or percentages were used to detail the values of each parameter in the overall group and the group of patients in the HGH group. In both groups, a Body Mass Index) BMI>25 is the most common comorbidity (36/45 of the overall cohort; 16/23 of the patients in the HGH group. HTN follows shortly as the second (25/24; 14/23). Diabetes mellitus falls third (20/45; 10/23), and cardiac abnormalities revealed by echocardiography are the least prevalent comorbidity in acromegaly patients (9/45; 3/23).

**Figure 2:**
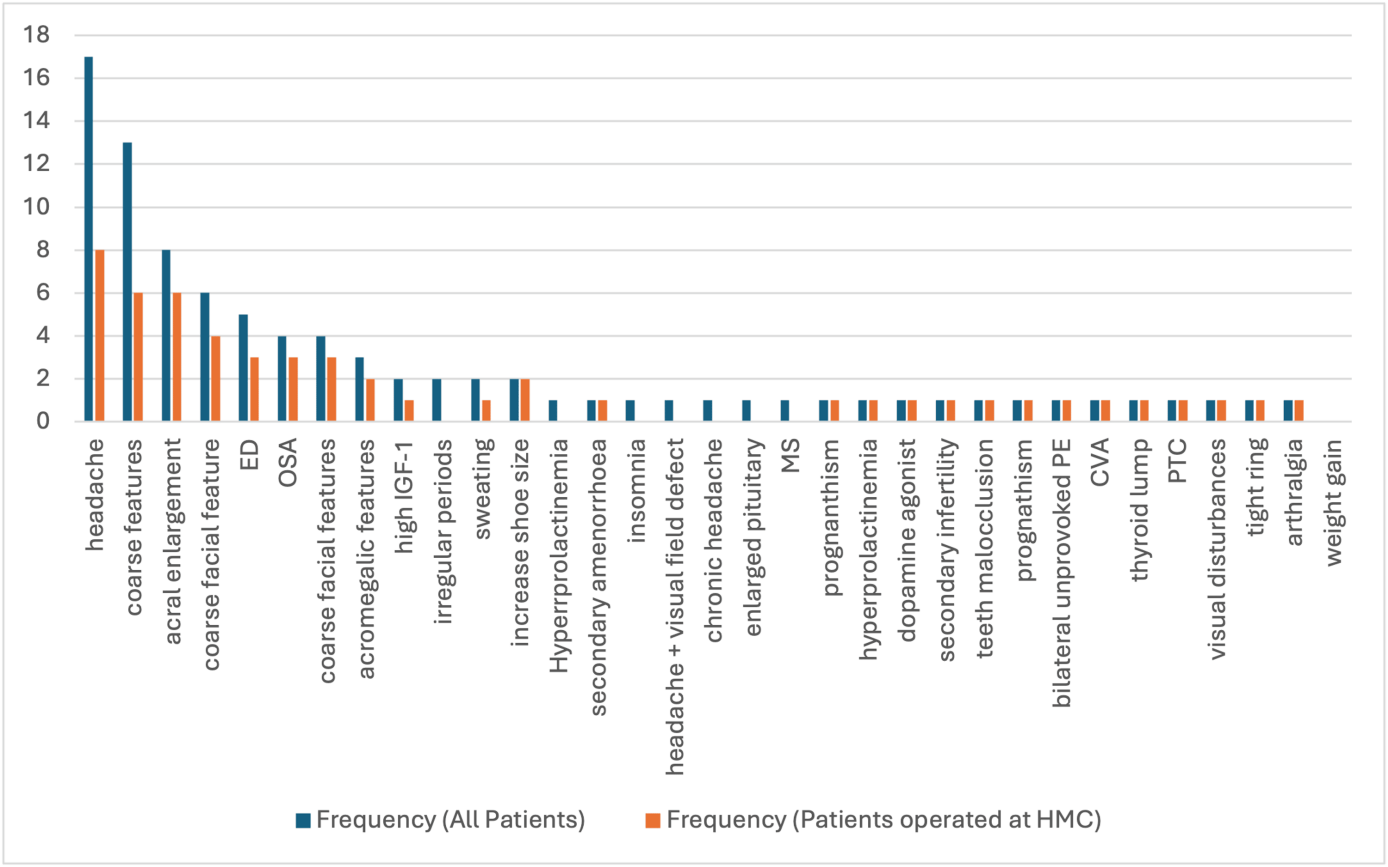
The frequency of various symptoms and diagnoses between all patients and those in the HGH group. HGH: Hamad General Hospital

Headache and coarse features were the most common symptoms in both groups, with a higher frequency observed in the overall patient population. In general, the HGH group presented with a lower frequency of most symptoms and diagnoses (Figure 2).

**Figure 3:**
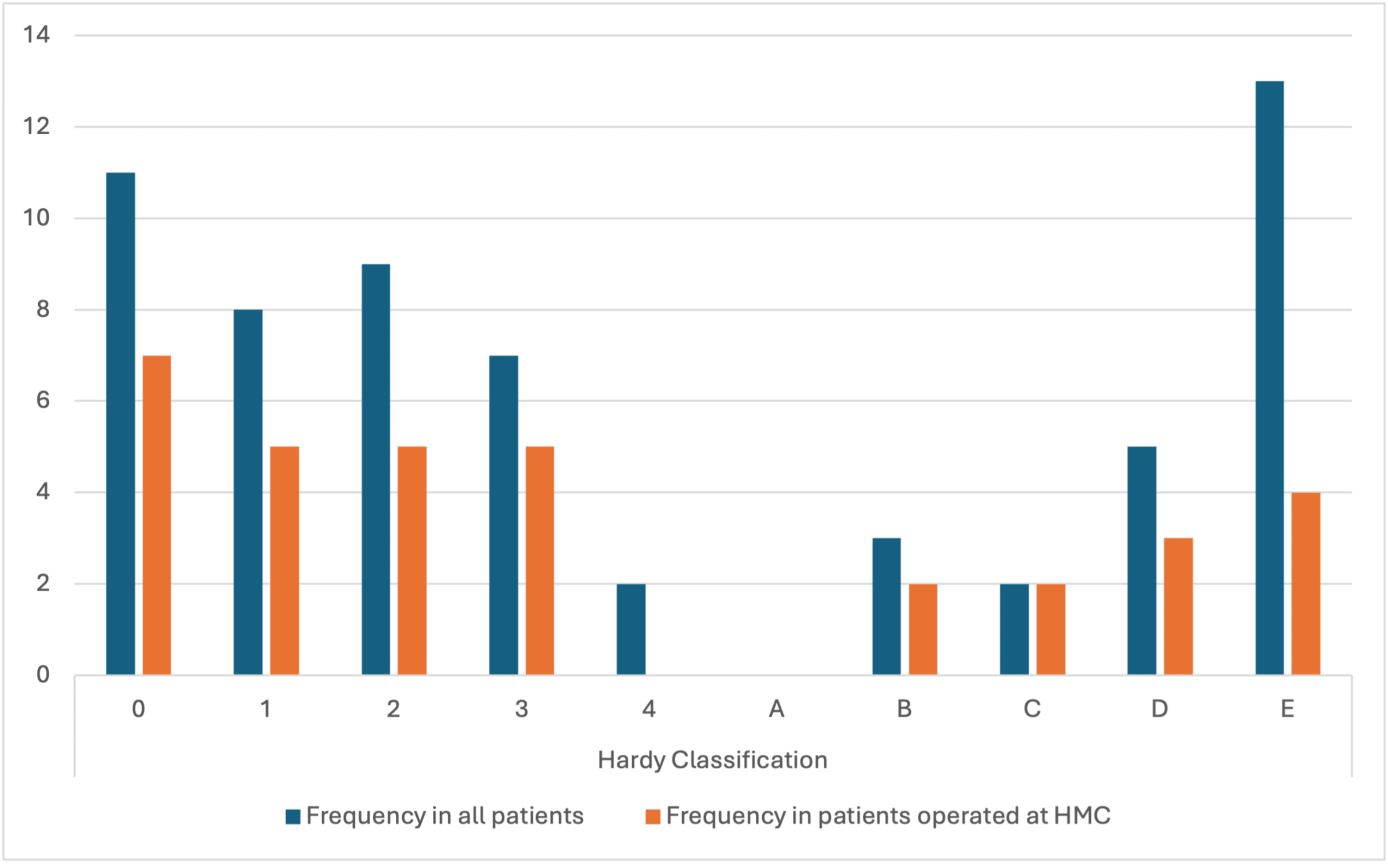
The distribution of patients across various Hardy Classification groups, comparing all patients to those in the HGH group. HGH: Hamad General Hospital; HMC: Hamad Medical Corporation

Hardy classification describes the morphological features of the adenoma on MRI to better localize the adenoma compared to gross localization. Most patients fall into the lower grades (0-3) and Grade E. Grade 4 is exclusive to the “all patients” group, with only two patients. No patients had a Grade A Hardy classification morphology (Figure 3).

**Figure 4:**
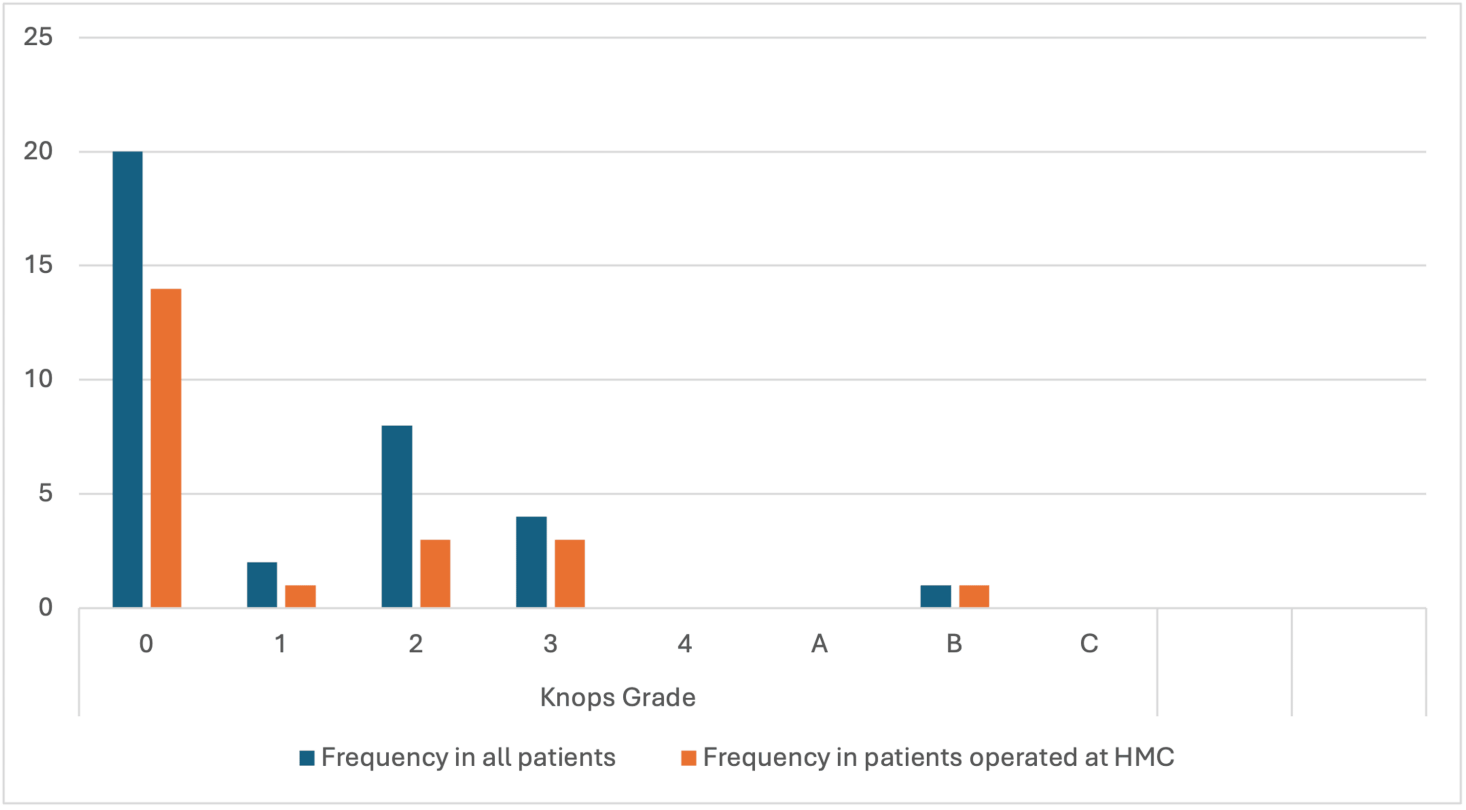
The distribution of patients across various Knosp’s Classification groups, comparing all patients to those in the HGH group. HGH: Hamad General Hospital; HMC: Hamad Medical Corporation

Knosp’s classification describes the invasiveness of the adenoma into the cavernous sinus on MRI (Figure 4). Grade 0 is the most prevalent in both groups, but the proportion is higher in the HGH group (60.9% vs. 44.4%). While Grade 1 proportions are similar, the whole cohort group has a higher proportion of Grade 2 (17.8% vs. 13.0%), and the HGH group has a slightly higher proportion of Grade 3 (13.0% vs. 8.9%). Grade 4, A, and C are absent in both groups, while Grade B, though rare, is proportionally higher in the HGH group (4.3% vs. 2.2%).

The percentage of patients showing an elevation in the gonadal hormone, ACTH, and TFT levels is similar among the patients of the overall cohort (20%, 15.5%, and 15.5%, respectively) and the patients receiving surgery at HGH (26%, 21.7%, and 17.3%, respectively).

**Figure 5:**
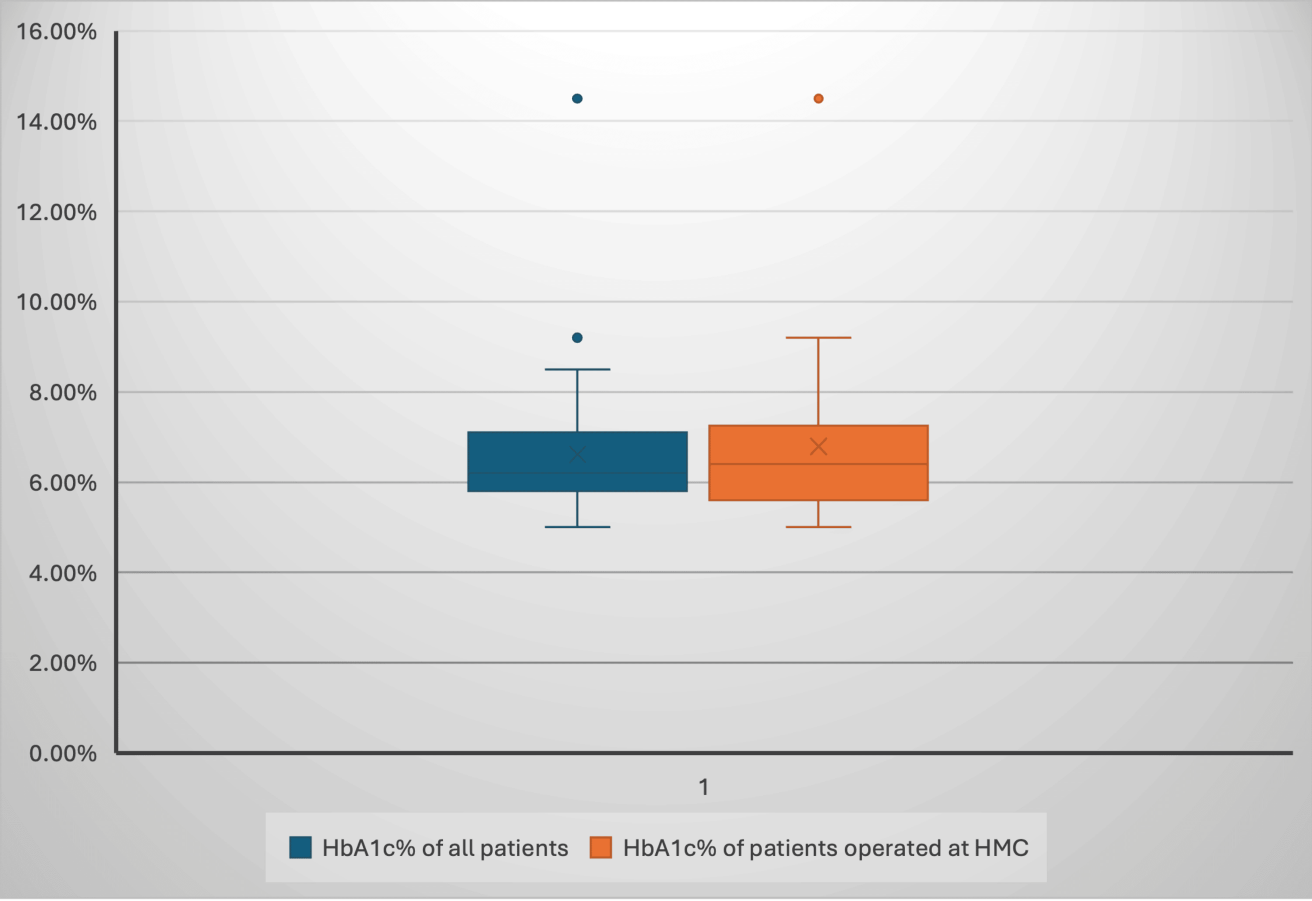
The box plot compares the distribution of HbA1c% levels between all patients and those in the HGH group. HGH: Hamad General Hospital; HMC: Hamad Medical Corporation

Both groups show similar HbA1c% distributions, with medians around 7.0% for all patients and 6.8% for HGH patients, suggesting comparable glycemic control between the groups (Figure 5). 

Endoscopic trans-nasal transsphenoidal surgery was the only surgical modality used for the treatment of all patients in the cohort. After surgery, the patients were given medical therapy. Some patients received radiotherapy. Slightly higher proportions of all patients compared to the HGH group received Octreotide and Cabergoline (42.2% vs. 39.1%, respectively, for Octreotide and 33.3% vs. 30.4%, respectively, for Cabergoline). Only six patients received radiotherapy, of which only one patient was operated on in HGH.

Postoperatively, pituitary function was assessed for all patients. A significantly higher proportion of HGH patients maintained intact pituitary function after surgery (73.9% vs. 60%), while fewer experienced hypopituitarism (26.1% vs. 40%).

**Figure 6:**
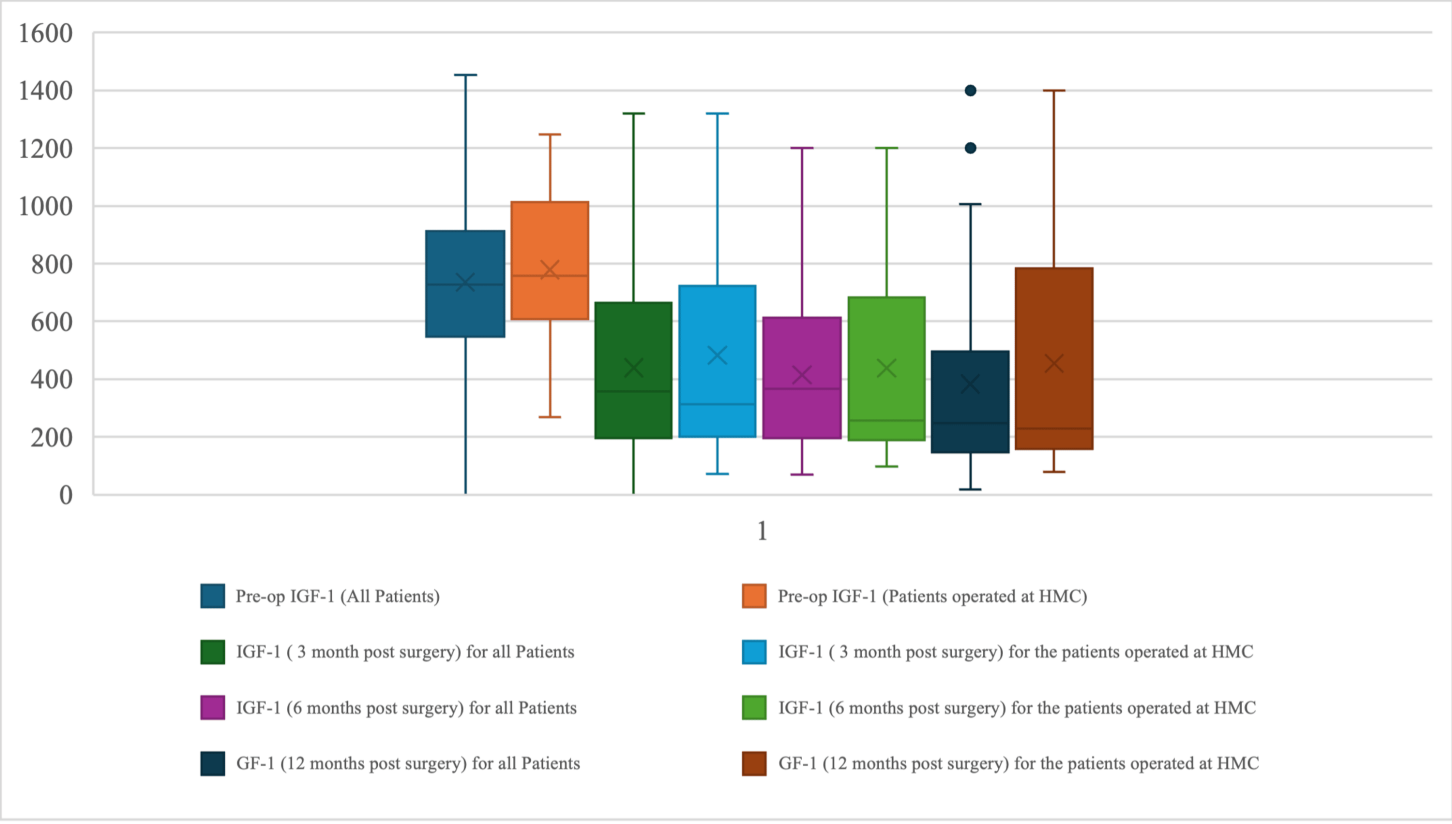
The variation of IGF-1 during the preoperative and the postoperative follow-up period until 12 months for the patients of the whole cohort and the HGH group. HGH: Hamad General Hospital; IGF-1: insulin-like growth factor-1

The variation of IGF-1 during the preoperative and postoperative follow-up period until 12 months was monitored for the patients of the whole cohort and the HGH group (Figure 6). Before surgery, the HGH group had higher IGF-1 levels (median ~850 ng/mL) compared to the overall cohort (median ~800 ng/mL). Following surgery, both groups experienced a significant reduction in IGF-1, with medians decreasing to approximately 450 ng/mL for HGH patients and 400 ng/mL for the overall cohort at 3 months post-op. These levels remained relatively stable throughout the 12-month follow-up period, indicating the lasting effect of the surgical intervention. Of note, the normal range for HGH is 56-120 ng/ml.

While the proportion of patients with residual tumors on MRI was similar between the two groups (25/45 overall vs. 13/23 at HGH), the recurrence rate was notably lower in the HGH group (4/23 vs. 9/45). The latter demonstrated a higher percentage of successful surgeries with no residue and no recurrence (43.4% vs. 33.33%). No HGH patients experienced recurrence when no residual tumor was observed on MRI. When residue was present, HGH patients had a higher recurrence rate (17.3% vs. 8.88%).

Overall, the “All Patients” group showed a lower prevalence of most findings, including GH (62.2% vs. 82.6%), PRL (24.4% vs. 26.1%), and Ki-67 1-2% (40% vs. 60.9%), except for Ki-67 2-3% (11.1% vs. 8.7%). Notably, FSH, α-subunit, and MEN-1 were not present in the patients in the HGH group. Inversely, "densely granulated morphology" was only present in the HGH group.

The postoperative complication rates between all patients and the HGH group showed that the latter group experienced lower complication rates. Subclinical hypopituitarism occurred in 2.2% of all patients and 4.3% of HGH patients, while subtotal resection was observed in 6.7% of all patients and none of the HGH patients. Diabetes insipidus was equally reported in all patients and HGH patients (4.4% and 4.3%, respectively). Hormonal replacement was necessary for 2.2% of all patients and 4.3% of HGH patients. CSF leakage, infection, pneumocephalus, and prolonged admission each occurred in one of the patients, but none of the patients in the HGH group.

**Figure 7:**
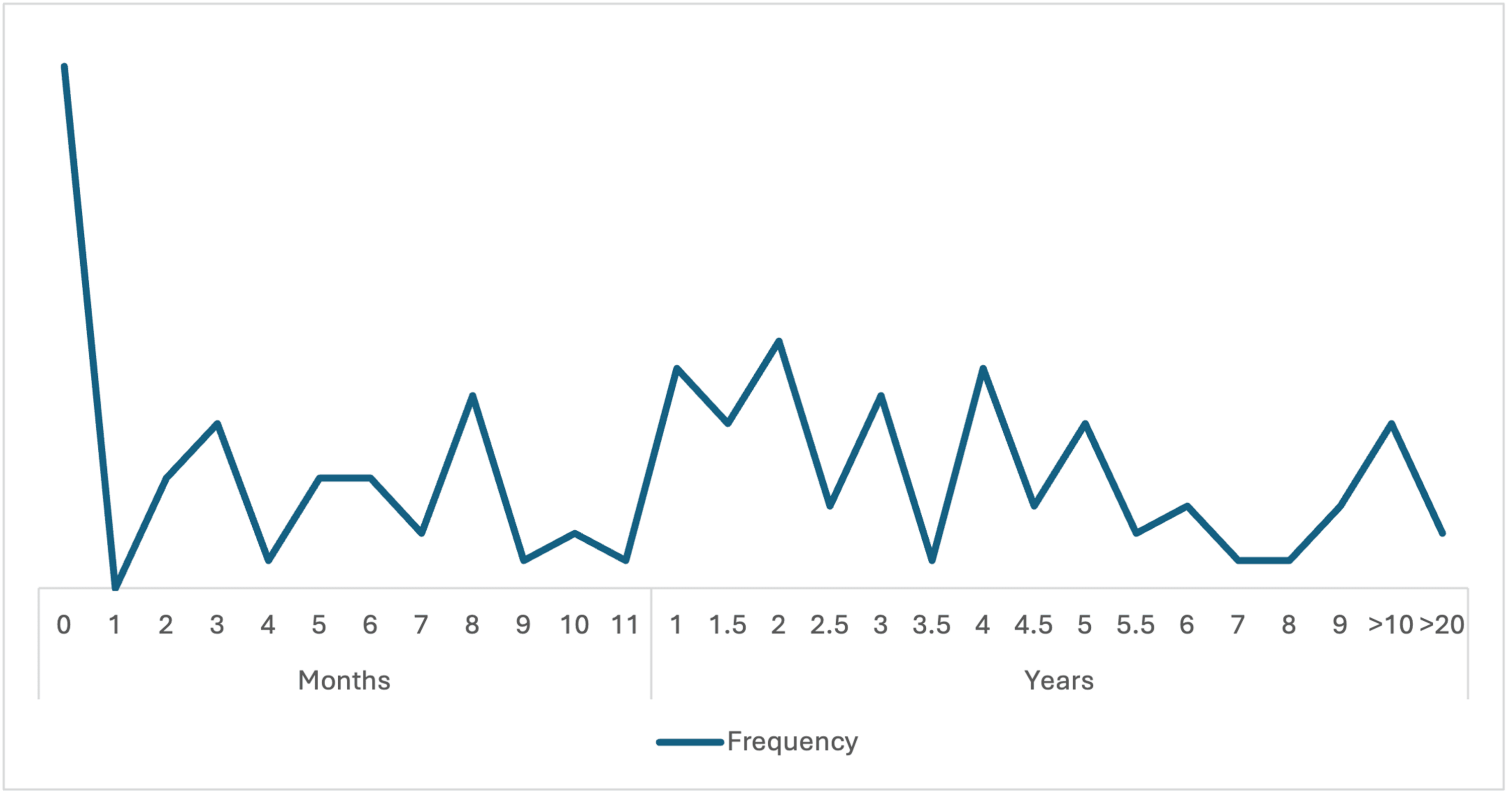
The longitudinal data on the frequency of MRI utilization following a surgical intervention. MRI: magnetic resonance imaging

The observed pattern in the longitudinal data on the frequency of MRI utilization reveals a distinct peak immediately postoperatively (month 0) (Figure 7). This is due to the routine immediate post-surgical assessment. However, fluctuations in imaging frequency persist throughout the first year, due to scheduled follow-up protocols. This oscillatory pattern extends into long-term follow-up, with notable peaks observed around the 2- and 2.5-year marks, and a low but persistent rate of MRI utilization even beyond 10 years post-surgery. The decrease in the frequency of MRI is anticipated because recurrence was limited to three cases only and was detected at 3 years after surgery. Hence, longer periods of observation and monitoring were implemented.

## Discussion

This study analyzes the demographics, clinical, radiological, and surgical characteristics of 45 patients with acromegaly, comparing patients who underwent surgical resection at HGH with those who underwent surgery at other institutions and were managed at HGH.

There was a similar demographic analysis in both groups with comparable median age and interquartile range. The small difference in mean age indicates that patients who had been operated outside HGH were slightly older. Analysis of gender distribution shows that males predominated in each group, though the proportion of females undergoing surgery at HGH was greater than that outside HGH. The largest subgroups were South Asians and Non-Qatari Arabs, along with the proportion of patients undergoing surgery at HGH differing by ethnic group.

The analysis of preoperative and postoperative data revealed that the most prevalent baseline comorbidity in acromegaly patients was concerning BMI> 25, followed by hypertension and diabetes mellitus. The lowest prevalence of cardiac abnormalities was found in the studied comorbidities. These results are inconsistent with previous research that shows that acromegaly patients experience elevated cardiovascular risk as a result of metabolic dysfunction [13,14]. Clinically, headache and coarse features were the commonest symptoms, with the overall cohort having a higher prevalence of these symptoms than the HGH-operated subgroup, which is in line with literature reports [7].

The radiological classification according to Hardy and Knosp’s criteria revealed a predominance of lower-grade tumors in both groups. Tumors of Grade 4 were seen only in the overall cohort, and no patients were classified as Hardy Grade A; on Knosp’s classification in patients with HGH, the frequency of Grade 0 was higher, whereas in this subgroup, Grade B occurred slightly more frequently. Our earlier findings are consistent with the literature suggesting that less invasive adenomas lead to better surgical outcomes [1].

The sole surgical approach used was endoscopic trans-nasal transsphenoidal surgery, followed by adjuvant medical and, in some cases, radiotherapy. Regarding endocrine evaluations, there were no significant differences in the respective postoperative levels of the gonadal hormones, ACTH, and TFT between the two groups, reflecting a homogenous effect of surgery on both groups. Additionally, there was no significant difference in glycemic control, determined using HbA1c%, between the two groups, indicating that diabetes management strategies yielded similar results. Indeed, prior studies have demonstrated that glycemic status is stable in postoperative acromegaly patients with well-controlled IGF-1 [4].

Overall cohort data indicated that, when compared with postoperative patients at HGH, significantly more patients developed hypopituitarism. Remarkably, much lower IGF-1 levels were observed postoperatively in both groups, illustrating that surgical correction was successful, and IGF-1 levels remained normal during a 12-month follow-up. These outcomes confirm the well-documented fact that IGF-1 normalization is one of the major predictors of successful surgical outcomes in the surgical management of patients with acromegaly [5].

Postoperative MRI findings were used to evaluate residual tumor and rates of recurrence. While the residual tumor was similar in both groups, the recurrence was significantly lower in HGH patients. This indicates that surgical outcomes were associated with a lower risk of tumor recurrence at HGH. Additionally, HGH patients showed higher rates of successful surgical outcomes, defined as no residual tumors or recurrence. However, in cases of residual tumors, the rate of recurrence was marginally higher in the HGH group. These results are consistent with some of the prior reports highlighting the need for early and accurate surgical intervention for lowering the risk of recurrence of the tumor [11].

On histopathology, postoperatively, GH positivity and Ki-67 1-2% were seen more often in HGH patients, while Ki-67 2-3% was more common in the cohort. Voting by receptor status analysis revealed the absence of some receptor findings in HGH patients (FSH, α-subunit, and MEN-1); nevertheless, densely granulated morphology was only present in this subgroup. It has been previously reported that patients with densely granulated tumors have a better surgical outcome than patients with sparsely granulated tumors in acromegaly [2].

All surgical complication rates were lower for the HGH group, with no instances of subtotal resection, CSF leak, infection, pneumocephalus, or prolonged admission. Diabetes insipidus and the requirement for hormonal replacement were seen with similar frequency in both groups. Previous studies have shown that validated surgical centers report lower complication rates than unvalidated centers, which can be ascribed to expertise in operative technique and perioperative care [13].

Within the postoperative period, we observed an expected upward pattern in the use of MRI, with the highest volume immediately after surgery, followed by the use of MRI for periodic assessment during the follow-up period. With only three recurrences detected at three years post-surgery, the decreasing frequency of MRI over time was justified; Indeed, the low recurrence rate noted, particularly in the HGH group. The frequency of follow-up MRI is consistent with the advice of the Endocrine Society guidelines on the management of acromegaly [10,15].

Limitations

The limitations of this study are summarized in Table 1. 

**Table 1:** Limitations of the study

Feature	Description	Implication
Sample Size	Small	Limits the generalizability of findings, making the statistical power low.
Design	Retrospective	Introduces biases such as missing or incomplete records and limits the ability to establish causality.
Single-Center	Bias	Generalizability of findings for other healthcare environments characterized by different surgical know-how, resources, and patient populations.
Surgical Management	Heterogenous	Surgical techniques and surgeon expertise may differ.
Follow-up period	Short	Not fully detect late-occurring attempts at tumor recurrence (after months and years) because of the nature of acromegaly-related tumors.
Histopathological Analysis	Variable	Different Ki-67 indices and receptor status among different groups, but the effect on clinical outcomes was not thoroughly analyzed.

Future implications

Future research should incorporate larger sample sizes and multicenter collaborations to improve the generalizability of findings and enhance statistical power. Engaging in prospective cohort studies or randomized controlled trials (RCTs) would allow for a reduction in retrospective bias and thus help clarify the cause-and-effect dynamics between surgical interventions and patient outcomes. Future studies should also involve multiple institutions, which would enable direct comparisons stemming from variations in surgical techniques, perioperative care protocols, and long-term patient-reported outcomes during follow-up to determine which practices are best. Follow-up beyond 12 months will allow a better assessment of tumor recurrence rates, late complications, and long-term efficacy of surgical and adjunctive treatments.

## Conclusions

Surgical management of acromegaly at HGH demonstrates favorable outcomes, with high rates of biochemical control, low complication rates, and good preservation of pituitary function. The incidence of recurrence was modest, and the majority of patients experienced meaningful clinical and radiological improvement following transsphenoidal surgery. These findings reflect the efficacy of a multidisciplinary approach to care, including preoperative endocrine optimization, advanced neurosurgical techniques, and structured postoperative follow-up.

Importantly, the data suggest that institutional surgical experience, continuity of endocrinological monitoring, and adherence to standardized follow-up protocols play a critical role in optimizing patient outcomes. The results underscore the value of maintaining high-volume centers of excellence for pituitary surgery in the region and support the need for ongoing investment in specialized care pathways for patients with pituitary disorders such as acromegaly.
